# p27^Kip1^, an Intrinsically Unstructured Protein with Scaffold Properties

**DOI:** 10.3390/cells10092254

**Published:** 2021-08-31

**Authors:** Debora Bencivenga, Emanuela Stampone, Domenico Roberti, Fulvio Della Ragione, Adriana Borriello

**Affiliations:** 1 Department of Precision Medicine, University of Campania “Luigi Vanvitelli”, 80138 Naples, Italy; debora.bencivenga@unicampania.it (D.B.); emanuela.stampone@unicampania.it (E.S.); fulvio.dellaragione@unicampania.it (F.D.R.); 2 Department of Women, Child and General and Specialist Surgery, University of Campania “Luigi Vanvitelli”, 80138 Naples, Italy; domenico.roberti@unicampania.it

**Keywords:** p27^Kip1^, intrinsically unstructured protein, scaffold protein, CDK, cyclin, cytoskeleton, Rho GTPase, αTAT1

## Abstract

The Cyclin-dependent kinase (CDK) regulator p27^Kip1^ is a gatekeeper of G1/S transition. It also regulates G2/M progression and cytokinesis completion, via CDK-dependent or -independent mechanisms. Recently, other important p27^Kip1^ functions have been described, including the regulation of cell motility and migration, the control of cell differentiation program and the activation of apoptosis/autophagy. Several factors modulate p27^Kip1^ activities, including its level, cellular localization and post-translational modifications. As a matter of fact, the protein is phosphorylated, ubiquitinated, SUMOylated, *O*-linked N-acetylglicosylated and acetylated on different residues. p27^Kip1^ belongs to the family of the intrinsically unstructured proteins and thus it is endowed with a large flexibility and numerous interactors, only partially identified. In this review, we look at p27^Kip1^ properties and ascribe part of its heterogeneous functions to the ability to act as an anchor or scaffold capable to participate in the construction of different platforms for modulating cell response to extracellular signals and allowing adaptation to environmental changes.

## 1. A Premise: Unfolding and Scaffolding Might Be Two Strictly Connected Features of Proteins

Different disciplines, like Mathematics, Biology, Genetics, Fundamental Physics, Astronomy and Philosophy, have pointed out the advantage of chaos as opposed to the Newtonian cosmos with its deterministic and linear order [1,2,3]. Indeed, the stochastic and chaotic features of life represent an immense advantage either for facilitating the long way of evolution or for allowing rapid environmental adaptations. In Biochemistry, the rigid protein structure/function paradigm has been progressively abandoned and, in the last twenty years, the discovery of proteins with extensive intrinsically disordered regions has gradually taken place. Partially or totally disordered proteins do not adopt a unique and rigid tridimensional structure and show a high grade of conformational plasticity. In turn, these apparently “chaotic” regions/proteins strongly increase the number of adaptive interactions extending the assortment of their molecular targets. Since ductility characterizes more than half of the human proteome, it can be reasonably assumed that IUPs/IUPRs (Intrinsically Unstructured Proteins/Intrinsically Unstructured Protein Regions) are involved in almost all basic and regulatory processes, accomplishing pivotal tasks in signal transduction, transcription, metabolic control and cell cycle [4,5,6,7]. IUPs, despite the lack of a 3D structure, are morphing over time and might transiently fold to adopt multiple conformations and to work properly. It has been argued that there is no direct evidence of their unstable state in the cellular environment, where, differently from the in vitro experimental conditions, the protein concentration is incredibly high. Thus, some concepts on IUPs that have found confirmation in vitro could not be completely reliable in vivo and IUPs plasticity could be, at least in part, hypothetical. Alternatively, it is conceivable that in vivo the different microenvironments of the various cellular compartments might provide specific interactors and conditions for proper folding in a process known as “folding upon binding” [8,9]. On these bases, the family of IUPs, rather than being defined as a pool of unstructured proteins, could be described as a class of macromolecules capable of rapidly challenging and modifying their three-dimensional architecture, thus undergoing a continuous refolding. This suggests, in turn, that the structure/function axiom may be still valid if adapted to the view that a single protein may give rise to multiple structure/function pairs, with even strongly diverging activities. 

To date, many methods, including complex bioinformatic assessments, confirm that the lack of stable protein organization is an intrinsic property of the proteome, with its levels increasing during evolution (>40% of the proteome in Eukarya branches) [10]. On these bases, a new proteome world has been proposed and called “disorderome”. Indeed, the impressive cell complexity in higher eukaryotes has been achieved using parsimonious strategies in which unfolded domains represent the most suitable method for network rewiring. Specifically, by means of modulable structural states of natively unstructured macromolecules, cells managed to use a limited set of proteins to carry out a fine control of different cellular processes. 

An extensive structural plasticity has also been observed in Scaffold Proteins (SPs) (also interchangeably defined Anchor, Adaptor or Docking Proteins) that play important roles in numerous cellular processes. Mostly, these proteins employ their modular structured domains, often in combination with the regions that do not assume stable secondary structures [11]. In the past, there have been many restrictions and limitations in recognizing and identifying novel SPs. The common mode of action of classical SPs involves their ability to connect functionally components of specific protein networks that act in signaling and/or metabolic pathways by increasing their effective concentration, or orienting the interacting partners in a conformation compatible with the cellular context, and/or by limiting their translational movements [12]. SPs are involved not only in signaling conduits, but also in assembly-line processes, in the control of enzymatic activities and in the transport of bound proteins (cargo function) to a precise cellular compartment [11,13]. They might also exert allosteric control over their partners and might be themselves the target of regulation. Overall, these definitions are unceasingly remolded, and new approaches are continuously introduced to predict candidate proteins which may act as scaffolds [14]. There is the possibility that anchor proteins may use both their structured domains and IUPRs in functional domain–domain interactions that could build up platforms and organizers of protein–protein interaction [15]. 

In the following, we aim to discuss experimental proofs sustaining the view that p27^Kip1^ (hereinafter, p27), a key cell cycle modulator and a well-recognized IUP, might be also envisioned as an SP.

## 2. p27, a Multifaced Growth-Modulating IUP

### 2.1. p27 as a CDK Regulator

p27 has been initially identified as a Cyclin-dependent kinase (CDK) inhibitor belonging to the CIP/Kip (Cyclin Inhibitory Protein/Kinase inhibitory protein) family. It plays fundamental roles in all phases of the cell division cycle. p27 is indeed a master regulator of G1 progression and G1→S transition and takes part in the control of G2 advancement and M phase/cytokinesis completion [16,17,18,19,20].

Mechanistically, the protein inhibits Cyclin E(A)/CDK2 and Cyclin A(B)/CDK1 by forming inactive ternary complexes and assists the physically assembling of Cyclin Ds and CDK4/6 heterodimers [17,21,22,23]. Because of these activities, the term CDK Regulator (CKR) may best apply to the p27 relations with CDK and Cyclins.

p27 has been one of the first IUPs whose mechanism of folding upon the binding has been characterized in detail. As matter of facts, biophysical and biochemical studies have allowed one to obtain a short but detailed movie that effectively showed the docking of the p27 N-terminal domain (including the kinase inhibitory domain or KID) on the Cyclin A/CDK2 heterodimer, characterizing the protein regions that bind, separately and sequentially, Cyclin A and CDK2 [24]. Precisely, the crystal structure of Cyclin A/CDK2/p27 displays an enlarged structural organization of the p27 KID, that consists of about 65 amino acids and embraces a rigid coil (named domain D1) which contains the cyclin-binding RxL motif, an amphipathic α-helix (i.e., LH), which spans the distance from cyclin A to CDK2, and a subsequent domain, defined domain D2, that binds CDK and includes an amphipathic β-hairpin, a β-strand and a 3_10_-helix. p27 is tethered to CDK2, producing a hybrid p27/CDK2 beta-sheet, with 3_10_-helix allocating into the catalytic cleft to block ATP access and, then, to inhibit the kinase. Through NMR analysis [25] and molecular dynamic simulation techniques [26], the precise mechanism of p27 freeing for CDK2 reactivation has been identified. Specifically, the phosphorylation of Tyr88 in the 3_10_-helix disrupts the inhibitory conformation of the hybrid beta-sheet. This allows structural changes in CDK2 to reestablish specific interfacial contacts with Cyclin A as in the active Cyclin A/CDK2 binary complex. Subsequently, the ejection of the 3_10_-helix is favored and the catalytic cleft of CDK2 becomes structurally very similar to that of the active Cyclin A/phospho-CDK2 complex. This is then able to lodge the ATP molecule as efficiently as the active holoenzyme, even without (Thr160) CDK2 phosphorylation carried out by CAK for the classical full activation [25,26].

These and other studies made progressively clear that p27, like its homologs p27^CIP1/Waf1^ and p57^Kip2^ (reviewed in [27]), has a versatile structure, that can fold piece by piece during the binding to the numerous different interactors. This process, however, can be seen as mutual, i.e., not only p27 folds upon binding to its partners, but also the interactors acquire a proper folding on p27. In brief, p27 might work similarly to an anchor and modulator of different proteins, thus displaying “scaffolding” activity.

The level and localization of either p27 or p27-interacting partners vary in different cell phenotypes and in relation to their functional state; this might be the basis of apparently conflicting findings on the outcomes of p27 level and subcellular localization. In addition, p27 structure and its interactions are controlled by an intricate network of post-translational modifications (PTMs), frequently, but nonexclusively, phosphorylation. The p27 unfolded state provides, like almost all IUPs, a very efficient housing for numerous PTMs. The importance of p27 PTMs has been evidenced in almost all functions and interactions as well as in the control of p27 localization, stability and degradation [28,29], thus rendering their spatiotemporal regulation a mechanism that deserves a great accuracy of investigation. Of note, PTMs can be also recognized as a key mechanism of regulation for SP activities.

### 2.2. p27 Is an Intermediate Component of Key Growth Regulatory Loops

Cells elaborate extracellular stimuli organizing an adaptive phenotype as an adequate response. This is necessary for the survival of living organisms [30]. The theoretical cell doubts: “Divide or not divide?” “Differentiate or remain undifferentiated?” “Survive or death?” (clearly, trivial paraphrases of a far more noble sentence) are solved through a strict control at crucial restriction points of cell cycle where signals conveyed by specific transduction pathways are elaborated. In this scenario, SPs might: (i) spatiotemporally regulate many signals, linking or localizing them into specific cell compartments; (ii) coordinate positive and negative feedback loops, and (iii) segregate in specific cellular compartments key pathway components. The Ras–Mitogen Activated Protein kinases (MAPK) is one of the major pathways for signal transduction from the plasma membrane to the nucleus and regulates the cell division cycle gears, controlling, among a plethora of targets, p27 level and localization. Indeed, following mitogenic stimuli and Ras-MAPK pathway activation, p27 is phosphorylated on Ser10 and conveyed from the nucleus into the cytosol [31]. Here, from being a target of phosphorylation, the protein might turn into a modulator of H-Ras localization and activity [32], unveiling a putative regulatory loop. In fact, cytosolic p27 reduces H-Ras activation by controlling its endosomal recycling and ubiquitination. Therefore, p27 elevates the mitogenic stimuli threshold needed for cell cycle reentry. In p27 knock-out mouse embryonal fibroblasts (MEFs), the monomeric GTPase showed an increased membrane localization and reduction in vesicle levels. Thus, in p27 KO cells, Ras activity is increased, due to its localization and altered mono or bi-ubiquitination [32,33]. Since the p27 KO phenotype was reverted by silencing stathmin, it has been suggested that p27 cytoplasmic localization and stathmin sequestration have crucial roles in this threshold control [32]. In brief, p27 prevents the full activation of H-Ras, controlling its trafficking in a stathmin-microtubule-dependent fashion and avoids an untimely cell cycle reentry, as a sensor and transducer of external stimuli intensity. The role of stathmin and microtubules in p27-dependent modulation of H-Ras activity suggest that the protein might act as a scaffold affecting cytoskeleton structure and its modulating/interacting factors. 

By using a similar strategy, p27 controls PI3K/AKT signaling by modulating its phosphorylated status. Growth-factor-induced PI3K activation results in phosphatidylinositol-3,4,5-triphosphate production, recruitment of AKT to the plasma membrane and subsequent AKT activation by two post-translational modifications. PDK1 and TORC-2 complex phosphorylate AKT, respectively, on T308 [34] and on S473 [35]. Fully activated AKT leads to a direct p27 phosphorylation (T157/T198), resulting in cytoplasmic sequestration and cell cycle progression in many cellular models [36,37]. More recently, p27 was identified as a positive regulator of the PHLPP2 (Pleckstrin homology domain leucine-rich repeat protein phosphatase 2) stability [38]. PHLPP2 has been considered a tumor suppressor that catalyzes the dephosphorylation of (phospho-S473)AKT [39]. Studying bladder cancer invasion, Peng et al. showed that p27 can stabilize PHLPP2 via inhibition of Calpain 1-mediated Hsp90 degradation, thus promoting the direct binding of Hsp90 with PHLPP2 protein for its stabilization. The negative effect of p27 on calpain-1 gene transcription appeared mediated by the attenuation of the Jak/Stat pathway [38]. These findings suggest a correlation between p27 and AKT phosphorylation in cancer invasiveness control, presenting the CKR as a coordinator of negative feedback signals in PI3K/AKT pathway. The details of this mechanism are still unclear and how p27 modulates Jak/Stat pathway deserve further investigations.

In summary, by acting either on CDKs or on CDK-independent transduction pathways, p27 is a general cell phenotype modulator, although not all mechanistic details of its actions have been clarified. p27 modular structure and the folding upon binding also suggest that this protein might be an anchor for different proteins (or protein complexes) and that this feature might help in explaining some of its still unclarified cellular activities.

## 3. p27, a Platform Where Assembling Different Complexes and Cellular Structures

Numerous pieces of evidence demonstrate that p27 is not simply a growth modulator. This activity might represent only one of the protein functions and probably additional roles still await to be identified. Mechanistically, a number of studies suggest that p27 could be envisioned as an SP favoring the interaction/modulation of additional proteins. The most straightforward example is its role in the assembly of early G1-Cyclin D/CDK complexes. Furthermore, in additional cases, p27 can be seen as a molecular platform for the recruitment of signaling proteins or enzymes involved in the bidirectional control of cellular processes. Following, we will show some instances suggestive of this view.

### 3.1. p27 and Cyclin/CDKs: Complex Formation and Control of Function 

**Figure 1:**
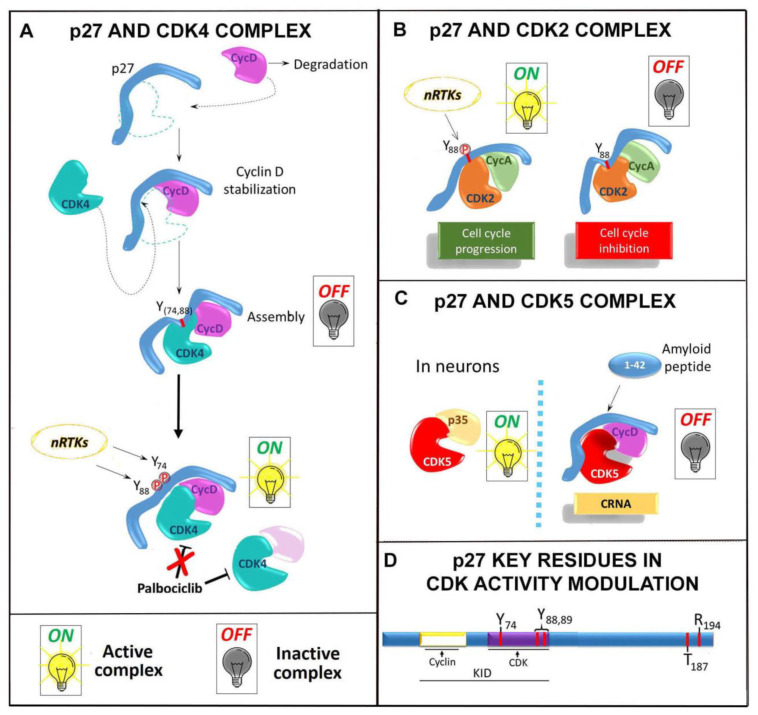
p27 and CDK interaction. The scheme summarizes some of p27 interaction with different Cyclin/CDK heterodimers. The sequence of the events is reported in detail in the text. (**A**) p27 binds sequentially Cyclin D and CDK4, facilitating the formation of the kinase complex. The modification of Tyrosine 74 (88) (P in red) by non-receptor tyrosine kinases (nRTKs) weakens the affinity of p27 for CDK4 which is then free to allocate the ATP in its specific ATP-binding site, allowing the full activation of the kinase complex. When bound to p27, the Cyclin D/CDK4 is resistant to inhibition by palbociclib, a specific Cyclin-D/CDK4 inhibitor now in clinical trials for high-risk ER^+^/HER2^−^ breast cancer. (**B**). p27 interaction with Cyclin A/CDK2 containing complex is recapitulated (see the text for further details). The panel highlights the binding of p27 KID to the Cyclin A and to the kinase CDK2 When working as inhibitor, p27 is strongly tethered to CDK2, with its 3_10_-helix allocated into the catalytic cleft of CDK2 to block ATP access and, then, to inhibit the kinase. The phosphorylation of Tyr88 in the 3_10_-helix disrupts the inhibitory interactions of p27 with CDK2 and allows the reestablishment of specific interfacial contacts between Cyclin A and CDK2, as in the active Cyclin A/CDK2 binary complex. Subsequently, the ejection of the 3_10_-helix is favored and the catalytic cleft of CDK2 becomes structurally very similar to that of the active Cyclin A/phospho-CDK2 complex. (**C**). The kinase CDK5 plays important roles in cell cycle arrest of postmitotic neurons. Its canonical interacting partner is p35 and when formed the complex p35/CDK5 is nuclear and active. In response to cell exposure to Aβ(1−42) peptide (an amyloid derived peptide), p27 seems to act as scaffold in favoring the association of CDK5 and Cyclin D1 in a trimeric complex with p27 itself that does not translocate in the nucleus, but hampers CDK5 interaction with its physiological partner and activator p35. This attenuates the CDK5 cell division restraining activity and might stimulate apoptosis. CRNA, cell cycle-related neuronal apoptosis. (**D**). p27 structure, with highlighted specific domains and amino acid residues playing a key role in the interaction with/activity modulation of CDK-containing complex. In the N-term moiety, the KID is highlighted with the amino acids target of phosphorylation (Y74, Y88, Y89) probably involved in the full activation of the kinase complexes. In the p27 C-term region, T187 represents the target of phosphorylation by CDK2 which addresses the protein to degradation; T157 and T198 phosphorylation by AKT may favor the assembly of Cyclin D/CDK4; R194 residue is also evidenced (at +7 from T187) which could allocate at the interface between Cyclin A and CDK2, forming specific hydrogen bonds with P272 on Cyclin A and E42 on CDK2 (see also the text for additional details).

p27 was identified as a Cyclin E/CDK2 interactor and inhibitor in TGF-beta arrested mouse epithelial cells [16]. Later on, p27 was surprisingly found in active CDK4 kinase complexes in proliferating cells [40], a finding that appeared contradictory to the previous studies. The incongruity was solved when p27 was characterized as an assembly factor promoting the formation of Cyclin D/CDK4(6) complexes during G1 phase (summarized in Figure 1A). As a matter of fact, in 1997, LaBaer and coworkers demonstrated that p27 allowed the assembly of Cyclin D/CDK4 complexes in vitro, suggesting that no other cellular activity was required for the assembly. Since Cyclin D1 and CDK4 lack nuclear localization signals, they also proposed that the anchorage of Cyclin D and CDK4 on p27 was an opportunity for both to enter the nuclear compartment and start the cell division cycle [41]. The observation provides the first evidence of p27 scaffolding activity. In addition to these results, the Pledger group, working on p27^−/−^ MEFs, showed that Cyclin Ds are quickly degraded and that the p27 reintroduction reversed the enhanced Cyclin D proteolysis and, in turn, induced the stabilization of Cyclin D/Cdk4 in ternary complexes [42].

Subsequently, Larrea and coworkers (2008) evidenced the p27 PTMs importance in these processes, confirming the relevance of PTMs in gaining scaffolding properties [43]. Like a classical anchor protein that uses many distinct domains to tether its partners, p27 uses key residues in its N-terminus (i.e., KID) and C-terminus to control CDK assembly and/or activity. p27 phosphorylations on T157 or T198 residues, due to AKT activity, appeared to promote the assembly of Cyclin D1/CDK4. Then, further phosphorylations of Y74 and Y88 (due to non-receptor tyrosine kinases) were required for the whole activation of the complexes. As a matter of fact, these tyrosine phosphorylations might decrease the inhibitory potential of the protein and allow Cyclin D/CDK4 complexes activation, despite being bound to p27 [23,44]. Very recently, Guiley et al. [45], by means of crystallographic studies, demonstrated that p27 binding causes a rotation of the N-lobe of CDK4 relatively to the C-lobe, determining the release of the activation segment from the active site of the enzyme and priming the enzyme to catalysis. Importantly, the full activation of the kinase activity is mainly determined by Y74 phosphorylation that weakens the affinity of p27 D2 domain for CDK4 which is then free to allocate the ATP in the active site. Therefore, p27 works as adaptor and allosteric activator of Cyclin D/CDK4 complex. Furthermore, the Cyclin D1/CDK4/pY74-p27 trimeric complex activity was shown to be independent of the presence of a docking site on the CDK4 canonical substrate pRb. The authors therefore suggested that a further role of phospho-p27 is to broaden the spectrum of CDK4 substrates in vivo. Finally, extremely important for clinical intervention is the demonstration that, when complexed with phospho-p27, cyclin D1/CDK4 complex is refractory to clinically relevant CDK4/6 inhibiting drugs such as palbociclib. Overall, these observations suggest that inhibition of p27 specific phosphorylations, cooperating with CDK4 inhibitors [46], could be a strategy to target CDK4-dependent tumors such as estrogen receptor–positive breast cancers. These data also emphasize the differences in signaling scenarios drawn by p27 versus phospho-p27, directly representing how different domains can be modified simply and speedily, to achieve different regulatory outcomes.

**Figure 2:**
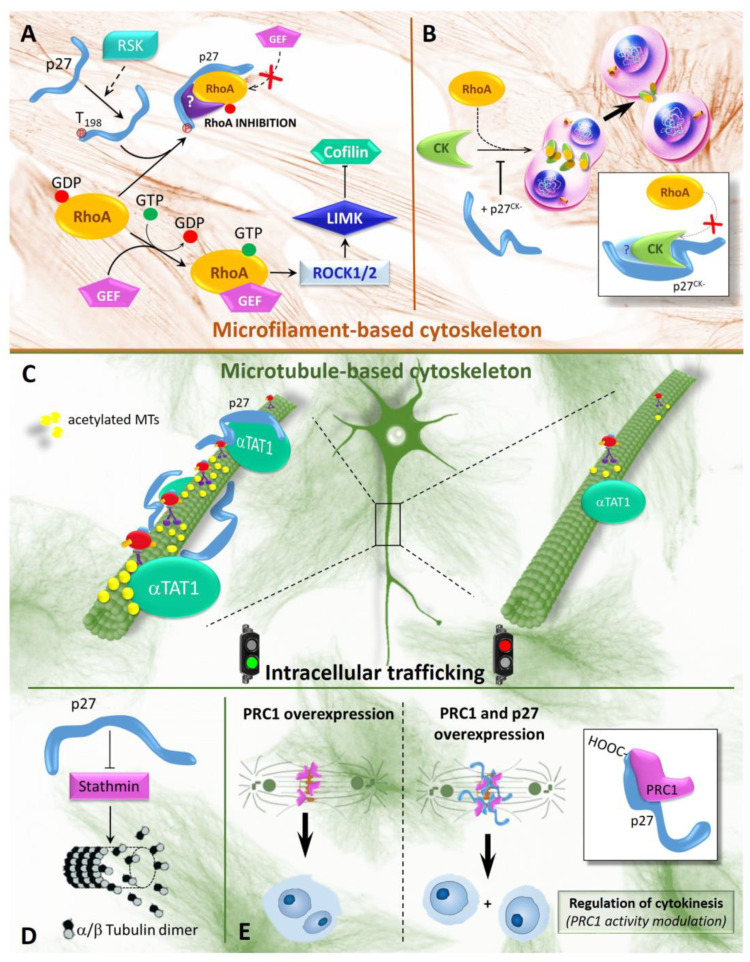
p27 and cytoskeleton remodeling. (**A**). In the cytoplasm, the monomeric G-protein RhoA bound to GDP is in an inactive state. Activation occurs through GDP-GTP exchange by a GEF (guanine nucleotide exchange factor). RhoA-GTP activates ROCK1/2, a kinase able to phosphorylate LIMK that subsequently phosphorylates and inactivates cofilin, a protein severing actin filaments. Therefore, Rho-A activation stimulates phosphorylation of cofilin and stabilizes the actin cytoskeleton. When phosphorylated by RSK1 (p90 ribosomal S6 kinase 1) on T198, p27 is mislocalized in the cytoplasm and is able to prevent RhoA activation by a still not clarified mechanism that could involve p115-GEF functional inactivation. Note that p27 region(s) involved in this activity are not definitely identified. RhoA inactivation results in full activity of cofilin and contributes to the loss of F-actin fibers and increased cell motility. (**B**). A further role of p27 during mitosis is independent of CDK control. p27 and p27CK^−^ (a mutant form of p27 unable to bind Cyclin-CDKs), when not T198-phosphorylated, are able to bind Citron Kinase (CK), preventing the interaction with its activators Rho. Citron Kinase is a serine/threonine kinase and is a Rho effector, with structural homology to ROCK, required in the ingression of the furrow cleavage (and midbody formation) for proper cellular abscission at the end of cytokinesis. p27CK^−^, competing with Rho for Citron K binding, mainly delay cytokinesis and also caused an increased reopening of the intercellular bridge at the end of cytokinesis, determining multinucleation. (**C**–**E**) This series of panels shows p27 and MT-dependent intracellular trafficking. (**C**) p27 controls the axonal flow of vesicles and organelles in isolated cortical projection neurons through modulation of acetylation of MTs. Mechanistically, p27 interacts, through its C-terminal mojety, with α-TAT1 **(**α-tubulin acetyltransferase 1), stabilizing the enzyme from proteasome-dependent degradation and therefore maintaining acetylated MTs and proper MT-dependent intracellular transport. In p27^KO^ neurons, α-TAT1 is degraded, MT acetylation is strongly down-regulated and there is reduced trafficking along MTs. (**D**). The panel schematizes the interplay between p27 and stathmin. In particular, it is shown that p27 might inhibit stathmin activity. Stathmin is required for microtubules depolymerization, probably acting either directly or sequestering the free tubulin heterodimers, and control of cell movement and plasticity. (**E**) The panel describes the interplay between p27 and microtubule bundling. p27 interacts with the microtubule-associated protein PRC1 (Protein Regulator of Cytokinesis 1), interfering with its ability to bind MTs and to cross-link antiparallel MTs. PRC1 acts at metaphase where it bridges kinetochore fibers, and later, at midbody, after cleavage furrow ingression. Thus, inhibiting PRC1 activity, p27 expression prevents the binucleation observed in PRC1 overexpressing cells.

Referring to the relations of p27 with Cyclin/CDK complexes, in addition to the mechanism of interaction of p27 with Cyclin A/CDK2 elucidated in detail (here described in paragraph 2.1 and summarized in Figure 2B), Li and colleagues [47] highlighted, mostly by bioinformatic tools, further regions, far-off the KID, that could be involved in Cyclin A/CDK2 stabilization into an active state and in proper substrate recognition, in both of which the R194-p27 (located at +7 from CDK2 phosphorylation site, i.e., T187) may play a crucial role. In detail, R194 seems to establish H-bonds with P272 of Cyclin A and E42 of CDK2, located at the binding interface between cyclin A and CDK2. The glutamate is conserved across many CDKs (CDK2, CDK3, CDK5 and CDK6) and lines in the extended loop of the PSTAIRE helix of CDK2, a segment that shows the most significant rearrangement upon Cyclin interaction [48]. In association with T160 phosphorylation, the PSTAIRE helix modulation leads to the realignment of the ATP-binding amino acids and p27 R194 interaction alters the position of the extended PSTAIRE loop, stabilizing the active cyclin A/CDK2 complex and probably enhancing its turnover rate. Among the consensus sequence of CDK2 substrates, R194 is found only in p27 (i.e., not in p21 and p57), suggesting that this is a p27 uniqueness and that the kinase has an exceptional effect on this protein that emerges as a key substrate in the control of cell cycle progression.

It is important to stress, however, that the importance of R194 has been experimentally validated by demonstrating that a peptide (180–194 of p27 sequence) including R194 is a better substrate of CyclinA/CDK2 than a similar peptide with alanine in 194 position [47]. Thus, further studies are clearly necessary for confirming the precise role of R194 in the ternary Cyclin/CDK/p27 complexes.

By virtue of its ability to control CDK interactors and CDK-containing complex formation, p27 also contributes significantly to neuronal apoptosis in response to neurotoxic agents, such as amyloid peptide Aβ(1–42) (Figure 1C). Many studies have suggested that CDK5, a neuron-specific CDK, may be important for cell cycle arrest of postmitotic neurons [49,50,51], and that Aβ(1–42) peptide, which alters the localization of CDK5, results in neuronal cell cycle reentry and cell cycle-related neuronal apoptosis (CRNA) [52,53]. It has been demonstrated that p27 promotes CRNA induced by Aβ(1–42), by stabilizing an atypical CDK5 complex, which results in kinase association with Cyclin D1 rather than with the canonical activator p35, resulting in attenuation of p35-associated CDK5 activity. As a result, neurons undergo cell cycle reactivation and consequent cell death [54]. Since the complex formed between these three proteins is significantly more stable than the binary complex, the occurrence of p27 possibly provides an explanation for Cyclin D/CDK5 aberrant activity despite the p35 presence, preventing the association of the kinase with its physiological partners and, then, its physiological roles. In this mechanism, we can clearly envisage p27 as SP (Figure 1C).

Focusing on its ability to establish distinct relationships with Cyclin and CDK partners, we could appreciate the exquisite p27 promiscuity in the pathogenesis of Primary Effusion Lymphoma (PEL) related to the Kaposi Sarcoma-Associated Herpesvirus (KSHV) infection. This oncogenic γ2-herpesvirus is considered as a carcinogen of class I by the World Health Organization for the high number of viral products that mimic human proteins, such as v-Cyclin, a viral cyclin homolog showing an elevated homology with human Cyclin D2. v-Cyclin binds to several CDKs and its interaction with CDK6 contributes to the pathological function [55]. It has been demonstrated that PEL-derived cell lines can express high levels of p27 while proliferating [56]. In 2004, Järviluoma and colleagues [57] also reported that v-cyclin binds to p27 in vivo, an interaction that contributes to the abrogation of its antiproliferative action. On the other hand, exogenous expression of p27 increases the levels of endogenous and transfected v-cyclin and CDK6. Importantly, these results also highlight a stabilization of v-Cyclin/CDK6 complexes, as expected in the light of the studies, at that time available, about p27 as mediator in the formation of the Cyclin D/CDK complexes. The same group successively demonstrated that in PEL cells p27, depending on the specific viral infection/replication phase, can form complexes with the v-cyclin alone, as already reported, and with the v-cyclin-CDK6 complex, which in this case shows reduced kinase activity. On the other hand, v-cyclin-CDK6 phosphorylates the bound p27 either on S10 and/or T187 to activate cytoplasmic relocalization or degradation, respectively, thus removing the inhibitory activity [58]. Finally, Figure 1D summarizes the key sites of p27 involved in Cyclin/CDK heterodimer assembly and modulation.

### 3.2. p27 as a Key Modulator of Cytoskeletal Dynamics and Cell Motility

Besides being a CDK regulator, p27 has been straightforwardly demonstrated to exert important roles in controlling cytoskeleton assembly/disassembly and cell movement. However, although the importance of the protein in these phenomena is functionally well established, the mechanistic details of p27 activity and its precise interactors do not appear definitely identified.

Three major classes of elements, differing in size and in protein composition, work together to form a cytoskeleton of eukaryotic cells [59]. Differently from intermediate filaments, made of different proteins, which have a very rigid structure inside the cells, actin microfilaments and microtubules have very dynamic structures. Actin filaments, organized in the form of meshworks or bundles of parallel fibers, contribute, together with the associated actin-binding proteins, to determine the cell shape and substrate adherence. Being continuously remodeled, they influence cell movements and cytokinesis during mitosis. Microtubules are highly dynamic long cytoplasmic polymers of tubulin [60] required for maintaining cell shape [61] and for the organization of mitotic spindle in dividing cells governing proper separation of sister chromatids and cytokinesis; they also are structural and functional constituents of cilia and flagella [62] and represent a structural platform for the intracellular movement of cellular organelles and for transport of vesicles. Furthermore, these structures are important for the assembly of macromolecules including enzymes and structural proteins and for facilitating the organization of pathways.

The initial indication that cytoplasmic p27 controls actin cytoskeleton affecting cell migration derives from the seminal study by Dowdy’s group published in 1998 [63]. The authors established that the migration of HepG2 cells (a human hepatocellular carcinoma cell line) treated with hepatocyte growth factor was dependent on p27 level. Particularly, they demonstrated that TAT-p27 protein transduction in HepG2 cells causes actin cytoskeleton rearrangement, formation of filopodia and cell migration. The authors also identified the domain involved in this activity in the C-terminal region of the protein. In 2003, the same group highlighted the importance of Rac1, a small GTPase, in this p27 scattering activity [64]. p27 intervention in the pathways modulated by the proteins belonging to the Rho (Ras homolog) family of GTPases, which includes Cdc42, Rac1 and RhoA (Ras homolog family member A), was further sustained in 2004 by the report of Besson et al. [65]. The study remarked behavioral defects in p27^−/−^ MEFs and suggested a molecular mechanism by which p27 regulates cell movement affecting activation of RhoA, thus enhancing the activity of several kinases strongly involved in cytoskeleton dynamics and cell motility [66]. One target of RhoA is ROCK (Rho-associated protein kinase) that phosphorylates/stimulates LIM kinase (LIMK). In turn, LIMK phosphorylates and inactivates cofilin. Since cofilin is an actin depolymerizing and severing factor, cofilin activity is central for cell motility [67,68].

According to the Besson’s study, p27 is able to block the binding of RhoA to its activators, the Rho–GEFs (Guanine nucleotide exchange factors) decreasing the accumulation of RhoA-GTP (Figure 2A). This induces, in turn, cell migration (due to reduction of phospho-cofilin) by mechanisms clearly distinct from p27 roles in cell cycle inhibition. The authors also identified in p27 C-terminus the protein region responsible for these effects. Recently, Phillips et al. [69] investigated the kinetics of p27 and RhoA interaction. They unexpectedly found that the direct binding between p27 and RhoA is remarkably weak and that, contrasting to previous data in the literature, p27 interferes with RhoA p115-GEF through its N-terminal half (aa 55–95 region) and not through the C-terminus. Considering that T198 phosphorylation boosts p27/RhoA binding [66], the authors also speculated the existence of intermediary partner(s) in p27/RhoA coupling, whose activity could depend on phosphorylation of the C-terminus p27 amino acid [69]. It is notable that a previous study reported that RSK1 (p90 ribosomal S6 kinase) might phosphorylate p27 on T198 and that this PTM promotes RhoA inhibition [70]. On these bases, the following possible sequence of events could be hypothesized. p27 interacts with RSK1 and is T198-phosphorylated. Then, the phosphorylated protein can act as anchor for RhoA probably through additional unidentified interactor(s) that interfere(s) with RhoA activation by p115-GEF. However, several other hypotheses could be suggested for explaining the findings in the literature and only direct evidence could clarify how p27 interferes with RhoA activity. In all the instances, the data by Phillips et al. [69] reinforce the view that several of p27 activities involve the formation of unidentified complexes facilitated by scaffolding function of the protein requiring (or not) PTMs. p27 also binds and reduces the activity of citron kinase, a pivotal modulator of cell abscission. This activity is probably due to a competition of p27 with RhoA, a major citron kinase activator. Importantly, citron kinase is able to interact and modulate a number of components of contractile ring and midbody and is required for a correct cytokinesis. Interestingly, both citron kinase and p27 have been demonstrated to colocalize at midbody level [20].

Focusing on the numerous additional aspects of the relationship between p27 and cytoskeletal dynamics, it is important to report its role in the regulation of neuronal migration and neurite branching, all mechanisms that entail actin and microtubule (MT) cytoskeleton remodeling. In this scenario, tubulin acetylation/deacetylation equilibrium is the protagonist, because it doses the molecular motors associated with microtubules and defines the velocity of organelle, protein or vesicle transport. Precisely, microtubule deacetylation causes locomotor deficits and disturbs MT-based axonal transport and neuronal connectivity [71,72]. Since the disruption of intracellular movement and exocytosis/endocytosis of synaptic vesicles have recently been demonstrated in a number of neurodegenerative diseases [73], the identification and the characterization of the mechanisms that regulate the process have also important clinical implications [74]. In a more recent study, it has been demonstrated that p27 controls transport along MT traces, governing the acetylation mechanism of alpha-tubulin. Accordingly, p27 depletion disrupts axonal flow in isolated cortical projection neurons without affecting MT polymerization [75]. Pretreatment of cortical p27 KO neurons with HDAC inhibitor tubastatin rescued axonal transport compared to the wild type counterparts. At a molecular level, p27 appears to decrease the degradation of α-TAT1, the α-tubulin acetyl-transferase that promotes MT acetylation and, hence, stabilization [75]. Surprisingly, this activity is carried out through its C-terminus, but does not require the critical residues (the 89–96 Proline-rich region) identified as harboring microtubule-binding activities for MT polymerization.

During neurogenesis, cell cycle exit, cell migration and neuronal differentiation must be harmonized and, apparently, they are linked by p27 activities played in proliferation control [76,77], MTs’ dynamic regulation [78] and stabilization of Neurogenin 2 [79]. The evidence of p27 N-terminal half in the Neurogenin 2 binding and stabilization represents an additional CDK- and cell cycle-independent activity played in the neuronal differentiation contributing to the cortex development and confirms p27 scaffolding role in the neurogenic program.

In summary, these data suggest the possibility that p27 can mostly work as an efficient scaffold macromolecule that allows not only the remodeling/interplay of the numerous cytoskeleton components but also that permits an exchange of functional information/regulation between cell division cycle machinery and cytoskeleton dynamics. As a matter of fact, MTs and actin filaments are intersected not only for cell motility, defining degree and direction, but also for cell polarity definition. This is of pivotal relevance in orienting cell migration and cell division in developing tissues. This modulation appears of particular importance during embryogenesis and neurogenesis, to form an organized and coherent pluricellular structure [80]. Additionally, a restricted window exists during mitosis, specifically at metaphase, in which MTs stillness negatively correlates with RhoA activity. While the plus– (+) end of some MTs binds kinetochores to be stabilized and the mitotic apparatus appears static, RhoA is inhibited in this limited period probably for cortical actin cytoskeleton reorganization and it reactivates only in anaphase and in restricted regions, for furrow ingression, which leads to cytokinesis. Despite extensive research, several questions concerning the regulation of cytokinesis remain unanswered and how the two cytoskeletons are linked is an open question: since the reported impact of Rho-GTPases on MT organization and dynamics (reviewed in [81,82]), it may be worth looking at p27 crucial GTPase-regulative activity to unveil one of the latent mechanisms of this crosstalk. 

It is interesting that p27 has also been reported to be a binding partner of an additional protein involved in MTs remodeling, namely, Protein Regulator of Cytokinesis 1 (PRC1). PRC1 is able to cross-link antiparallel MTs and plays a key role in central spindle formation. p27 interferes with the activity of PRC1 to bind MTs, thus hampering the increased MT bundling due to PRC1 overexpression [83].

Two further components of the complex relationship between p27 and cell movement should be taken into consideration, namely, the interaction at cytosolic level of the protein with the MT-destabilizing protein stathmin and the ability of the protein to bind cortactin. In the first instance, p27 interferes with the stathmin capability to modulate MTs during the interphase, hampering their remodeling. In this case, p27 appears to reduce cell movement [84]. In the second condition, p27 promotes the interaction of cortactin with PAK1 (p21(Rac1) activated kinase), a kinase activated by Rac1, thus clearly acting as scaffold. Activated PAK1, in turn, allowed invadopodia turn-over [85]. 

In summary, p27 appears to interact with numerous distinct cytoskeleton-correlated proteins, RhoA, p115-GEF, PRC1, Citron-K, αTAT1, RSK1, cortactin and stathmin. In only one case, the binding affinity (by equilibrium dissociation constant, K_d_) has been investigated in details (p27/RhoA) and the results have suggested that the interaction is probably indirect. The same probably occurs in other cases, and p27 might represent an SP that allows complex interactions. It is to be underlined that several of the proteins reported above as p27 interactors are also able to interact with each other, suggesting the formation of dynamic complexes able to rapidly change their compositions/function similarly to DNA interacting complexes. Thus, although the relevance of p27 in cytoskeleton dynamics and function has been widely confirmed, additional structural/functional studies appear absolutely compulsory to shed light on this complex scenario. In Figure 2, the main p27 activities on the cytoskeleton are summarized.

### 3.3. p27, Lysosomal Function and Autophagy

Several lines of evidence have associated p27 and autophagy activation under conditions of stress and nutrient deprivation [86,87,88,89,90]. Interestingly, as demonstrated very recently in glucose-deprived MEFs, the autophagosome trafficking inside the cells towards the perinuclear area, where the lysosomes are generally concentrated, occurs along MTs and is controlled by p27 that maintains a high level of MT acetylation through stabilization of αTAT1 [72]. This finding extends the cytoplasmic roles of p27 linking the CKR activities at the crossroad among cytoskeleton regulation, cell motility and resistance to stress conditions such as nutrient deprivation. Strikingly, a recent report of Besson’s group further investigates the p27 activities in stimulating the autophagic process during prolonged amino acid starvation [90,91]. Particularly, the authors showed that p27 interacts with LAMTOR1, an important component of the so-called RAGULATOR complex involved in the control of mTORC1 activity. The RAGULATOR complex is made of 5 subunits, LAMTOR1, 2, 3, 4, 5 with LAMTOR1 exerting scaffolding activities and allowing lysosome anchoring and formation of the mature complex. Once organized, RAGULATOR aggregates two GTPase dimers, which, in turn, are able to recruit mTORC1 to the lysosome where it will become activated by interaction with Rheb [87,88,89].

When p27 binds to LAMTOR1, it impedes the scaffold activity of the protein in building the RAGULATOR complex, thereby affecting negatively mTORC1 activity. This also determines that the transcription factor TFEB, which is normally phosphorylated by mTOR (the kinase component of MTORC1) and sequestered in the cytoplasm, is freed of translocating into the nucleus, stimulating transcription of several genes involved in autophagy and lysosome recycling. In this molecular mechanism, although p27 does not act as a classical scaffold, it interacts directly with the lysosome SP LAMTOR1, hampering its activity and affecting the formation of a highly structured complex. On the other hand, we cannot exclude that p27-LAMTOR1 interaction might represent the initial base of an alternative complex still uncharacterized. In this scenario, it may be interesting to underline that p27 binding affects the GAP (GTPase activating proteins) activities of LAMTOR1, previously identified as p27RF-Rho (p27^Kip1^ Releasing Factor from RhoA). By impeding the RAGULATOR complex formation, p27 also hinders the recruitment and consequent activation of key GTPases such as Rag and Rheb (the latter acting on MTORC1 for its full activation). Remarkably, this GTPase-targeting activity seems a general mechanism of action of cytoplasmic p27 in affecting cytoskeleton dynamics, organelle and vesicle movement, autophagy and cell motility.

### 3.4. p27 as a Platform for Transcriptional Complexes

Recently, a body of experimental evidence suggest that p27 might act as modulator of transcription. The topic has been exhaustively described and discussed in detail in recent reviews and we refer readers to the citations contained therein for further details [92,93]. p27 might control gene expression, acting as coregulator of c-Jun. Structurally, the interaction appears to involve the C-terminal region of p27 [94]. As above reported, this part of the protein is subject to numerous phosphorylations that, in turn, could regulate the interaction with the transcription factor. Thus, at least in theory, the phosphorylation of p27 could represent a mechanism for connecting the control of specific kinase activity and gene expression. Mechanistically, the interaction with phosphorylated p27 enhances the recruitment of c-Jun on target genes activating oncogenic pathways and, contemporaneously, down-regulating differentiation mechanisms. In addition to interacting with c-Jun, p27 plays the role of putative corepressor with p130, E2F4, HDAC1 and SIN3A [95,96,97]. Further transcription factors that have been suggested to bind p27 are STAT3, MyoD, AHR, PAX5, PCAF1 and NGN2 [92,93]. A detailed description of p27 transcriptional phenotypical effects is out of the scope of this review; however, two points, in our view, need to be underlined. The first is that it is not clear whether the transcriptional activity of p27 is strictly or partially dependent on its activity as CDK regulator. The second is mainly related to the focus of this review. It is now definitely clear that the modulation of transcription requires the dynamic and rapidly reversible formation of different protein complexes that act not only on the regulatory regions of the genes but also on the DNA accessibility. In these complexes, a number of components play the role of adaptors. Under this view, it may be hypothesized that, at least under specific conditions, the activity of p27 on gene expression could be to facilitate the formation of gene-interacting complexes. In other words, p27 might be a scaffold component of transcriptional complexes and, by this activity, could act as a transcriptional modulator.

## 4. Perspectives and Conclusions. p27, Human Diseases and Drug Design

In the past years, compelling evidence has demonstrated the importance of p27 (both as level and activity) in numerous human diseases, mainly in human cancers. The first observation of a possible role of p27 in cancerogenesis stems from a seminal study on mice ablated of the *Cdkn1b* gene [72]. These genetically modified animals, in addition to showing an increased body size and hyperplasia of several organs, developed pituitary adenomas [72]. Furthermore, *Cdkn1b*^+/−^ mice were more susceptible than normal animals to the development of cancer due to chemical carcinogens or irradiation [98]. Some years later, it was shown that rats with a homozygous alteration of *Cdkn1b* had a peculiar form of MEN, called MENX [99]. Pellegata and collaborators have also described the spontaneous development of MENX in rats hemizygous for *Cdkn1b* although with a lower frequency with respect of homozygous animals [100]. The finding strongly reinforces the view of *Cdkn1b* as a haploinsufficient tumor suppressor gene in mouse and rats. Numerous findings have also corroborated the opinion that p27 alterations are important in human carcinogenesis. Indeed, p27 decrease has been demonstrated in breast, colon, prostate and ovarian cancers [27]. Moreover, tumors with p27 reduction also showed increased aggressiveness. The molecular basis of p27 decrease has been, at least initially, associated with an enhanced degradation or with a mislocalization of the protein in the cytosol [27]. More recently, due to the development of novel technological approaches, i.e., Next Generation Sequencing and Genome-wide Association Analyses, *CDKN1B* has been identified as mutated in several cancers. In luminal breast cancers, *CDKN1B* constitutes one of the 18 genes most frequently altered [101]. Moreover, in this frequent breast cancer type, the downregulation of p27 causes resistance to anti-HER2 therapies and radiotherapies [102]. *CDKN1B* has also been found frequently altered in prostate cancers and in hairy cell leukemia where it represents the second most altered gene after BRAF [103]. Finally, in sporadic parathyroid adenomas, in small intestine neuroendocrine tumors [104,105,106] and in MEN4 (a new MEN subtype), *CDKN1B* is recurrently altered [107]. All these finding definitely demonstrated a role of p27 in human cancerogenesis and/or in tumor evolution.

p27 alterations have also been demonstrated in a specific condition associated with human diseases. Indeed, in endometriosis, p27 appears significantly reduced, and in congenital eye alteration, p27 post-synthetic changes have been demonstrated [108,109]. Haploinsufficiency of *CDKN1B* was demonstrated to improve cardiac function in mice post-myocardial infarction [110]. Finally, a role of p27 in subarachnoid hemorrhage has been reported [111]. Based on all this evidence, the possibility of targeting p27, either positively or negatively, appears of pharmacological interest. 

In the development of novel pharmacological therapies, among the properties that an ideal drug should have, a high selectivity is certainly of fundamental importance. Therefore, the plasticity of IUPs and their large number of interactors would suggest that these proteins might not be therapeutic targets. This view is also supported by the obligatory absence/scarcity of IUPs crystallographic data that, conversely, are of great relevance in drug design [112]. 

Recently, however, several different studies have demonstrated that an unfolded protein or unfolded protein region might be targetable for therapy [113]. For example, other IUPs, namely EWS-FLI1 (a fusion oncoprotein involved in Ewing’s sarcoma), c-Myc, Alzheimer β-amyloid peptide and α-synuclein, appear to be druggable by small molecules or peptides [114,115,116,117,118,119,120]. Interestingly, the binding between p53 and MDM2 might also be altered even if p53 has also been demonstrated to be an IUP. In the last case, the treatment of cells with specific peptides resulted in an increase of both p53 and MDM2 and in the activation of an antiproliferative program [121,122].

A recent study by Kriwaki’s group reported the possibility of affecting the binding of p27 with other proteins [123]. The study was performed with the goal of reducing p27 cytosolic carcinogenetic properties. Through NMR-based screening, small molecules that bound p27 specifically, albeit with low affinity, in regions containing aromatic rings (with predilection for amino acids W60/76 and Y88/89) of the kinase inhibitory domain were identified. The authors also demonstrated that one of the tested compounds sequesters p27 from Cyclin A/CDK2, binding its D2 domain, a p27 region that specifically interacts with CDKs and folds upon this binding. The drug-dependent p27 displacement restores CDK2 catalytic activity, demonstrating the pharmacological effect of this small molecule, able to block p27 function. Despite this effect being associated with cancer development, the proposal of the Kriwacki group demonstrated how it will be possible to drug transient unfolded protein-folded protein interfaces [123]. 

Why are these studies promising? Since p27 might be considered in some instances an SP, the Kriwaki study suggests that proteins facilitating the formation of complexes, albeit unstructured, might be the target of therapy, allowing the conclusion that the knowledge of the region involved in specific binding, even if part of IUP, might be considered for efficacious therapy. Obviously, their relevance depends on a detailed knowledge of interactors of the protein region to be targetable. 

A further aspect should be underlined. p27 decreased level, cellular mislocalization and structural changes due to mutations are all events correlated with human diseases. In this context, the ability of the protein to form different complexes acting as anchor should be investigated to unveil more comprehensive pathogenic mechanisms and to develop more efficacious therapeutic approaches. As frequently in Science, although much is known, the complete picture remains to be understood. Accordingly, in our view, the importance of randomness in biological and clinical phenomena must never be forgotten.
